# Oxygen-Induced Retinopathy from Recurrent Intermittent Hypoxia Is Not Dependent on Resolution with Room Air or Oxygen, in Neonatal Rats

**DOI:** 10.3390/ijms19051337

**Published:** 2018-05-01

**Authors:** Kay D. Beharry, Charles L. Cai, Jacqueline Skelton, Faisal Siddiqui, Christina D’Agrosa, Johanna Calo, Gloria B. Valencia, Jacob V. Aranda

**Affiliations:** 1 Department of Pediatrics, Division of Neonatal-Perinatal Medicine, State University of New York, Downstate Medical Center, Brooklyn, NY 11203, USA; 2 Department of Ophthalmology; State University of New York, Downstate Medical Center, Brooklyn, NY 11203, USA; 3 Departments of Pediatrics & Ophthalmology, State University of New York Eye Institute, New York, NY 10062, USA

**Keywords:** angiogenesis, astrocytes, insulin-like growth factor-I, intermittent hypoxia, oxygen-induced retinopathy, retina, vascular endothelial growth factor

## Abstract

Preterm infants often experience intermittent hypoxia (IH) with resolution in room air (RA) or hyperoxia (Hx) between events. Hypoxia is a major inducer of vascular endothelial growth factor, which plays a key role in normal and aberrant retinal angiogenesis. This study tested the hypothesis that neonatal IH which resolved with RA is less injurious to the immature retina than IH resolved by Hx between events. Newborn rats were exposed to: (1) Hx (50% O_2_) with brief hypoxia (12% O_2_); (2) RA with 12% O_2_; (3) Hx with RA; (4) Hx only; or (5) RA only, from P0 to P14. Pups were examined at P14 or placed in RA until P21. Retinal vascular and astrocyte integrity; retinal layer thickness; ocular and systemic biomarkers of angiogenesis; and somatic growth were determined at P14 and P21. All IH paradigms resulted in significant retinal vascular defects, disturbances in retinal astrocyte template, retinal thickening, and photoreceptor damage concurrent with elevations in angiogenesis biomarkers. These data suggest that the susceptibility of the immature retina to changes in oxygen render no differences in the outcomes between RA or O_2_ resolution. Interventions and initiatives to curtail O_2_ variations should remain a high priority to prevent severe retinopathy.

## 1. Introduction

Retinopathy of prematurity (ROP) is a neovascular disease causing childhood blindness worldwide [1]. ROP has significant deleterious impacts on other ocular diseases—such as glaucoma, amblyopia, strabismus, myopia, and retinal detachment [2,3]—and huge financial and emotional burdens to families and societies [4,5,6,7]. ROP is a developmental vascular disorder caused by abnormal growth of retinal blood vessels in the incompletely vascularized retina of extremely low gestational age neonates (ELGANs) who are <28 weeks gestation [8,9,10,11]. The etiology of ROP is multifactorial, involving exposure to supraphysiologic oxygen and resulting complex pathophysiologic events of oxidative distress, inflammation, poor nutrition and dysregulated growth factors, and intermittent hypoxia (IH).

Neonatal IH—defined as brief, repetitive cycles of arterial oxygen desaturations followed by reoxygenation [12,13,14,15,16]—has emerged as one of the major factors associated with severe ROP in ELGANs requiring oxygen therapy [17,18,19,20,21,22,23]. An IH event is usually defined as a decline in SaO_2_ by 5% lasting <3 min in duration [13,14,15]. Reoxygenation following an IH event can occur in normoxia or hyperoxia (Hx), but whether the effects of IH with resolution in normoxia is less injurious to the immature retina than that in Hx remains to be determined. ELGANs experiencing the highest incidence of destaturations, bradycardia, and apnea were more vulnerable to malnutrition, extrauterine growth restriction, and nutrient deficits [24], associated with persistent low serum insulin-like growth factor (IGF)-I, which, together with vascular endothelial growth factor (VEGF), plays a crucial role in normal and aberrant retinal angiogenesis and vascular development [25,26,27].

Angiogenesis is regulated by complex interactions between locally and systemically produced growth factors that influence differentiation, proliferation, migration, and intrusion of endothelial cells, ECs [28]. VEGF is the most dominant attractor for ECs, and the magnitude of attraction varies depending on its isoform and binding capacity. The VEGF gene consists of 8 exons separated by 7 introns [29]. The gene is spliced to form multiple isoforms of varying amino acids according to the presence or absence of exons 6 and 7, which encode heparin-binding domains [30]. Of the many isoforms of VEGF identified, VEGF_165_ (VEGF_164_ in rodents), is the most abundant, has a single heparin-binding domain with basic residues encoded by exon 7, and has an intermediate affinity for the extracellular matrix [31,32]. The VEGF gradient provides a chemical cue to promote the speed and motility of ECs [33,34]. Angiogenesis that drives normal retinal vascularization begins at approximately 17 weeks of gestation and is complete at term [35]. It involves “physiologic hypoxia” when the metabolic demand of the maturing retina increases but is not sufficient to supply the underlying choroid and the growing vascular network [36]. In response to “physiologic hypoxia”, VEGF is secreted predominantly by glial cells (astrocytes and Müller cells), stimulating new vessel formation [37]. Unlike Müller cells, which extend from the inner limiting membrane (ILM) to the outer limiting membrane (OLM), with their end-feet residing in the nerve fiber layer (NFL)/ganglion cell layer (GCL), astrocytes are confined to the NFL/GCL and are intimately associated with retinal blood vessels [37,38,39,40,41]. This restriction to the inner retina enables the astrocytes to rapidly respond to hypoxia with resulting increased expression of VEGF and vessel formation [37]. Avascular retinas contain no astrocytes, while retinas that are highly vascularized contain diffusely distributed astrocytes [41]. Degeneration of retinal astrocytes in models of oxygen-induced retinopathy (OIR) results in blood–retinal barrier failure [42] and retinal detachment [39].

The retinal vasculature is highly reactive to changes in oxygen tension [43]. Hx suppresses vessel formation in the developing retina and VEGF expression by astrocytes and Müller cells [37], while hypoxia induces VEGF and neovascularization. Several animal models for ROP have been developed utilizing the Phase 1 (vaso-obliteration)/Phase 2 (vaso-proliferation) hypothesis originally proposed by Ashton et al. in the 1950s [44]. However, many of these models fail to incorporate the features of recurrent neonatal IH experienced by extremely immature neonates born at <28 weeks gestation, who are at the highest risk for severe ROP. Our rat model of neonatal IH-induced retinopathy has repeatedly produced many characteristics consistent with severe ROP, including vascular tufts, neovascularization, hemorrhage, dilated and tortuous vessels, photoreceptor degeneration, choroidal ruptures, and, more recently, astrocyte and Müller cell activation [45,46,47,48,49,50,51,52,53]. This model, which recapitulates frequent, brief, arterial oxygen desaturations experienced by ELGANs, showed that neonatal IH induces mechanisms that regulate both Phase 1 and Phase 2, within minutes of each other, with minimal recovery time between phases. The mechanisms that regulate Hx and hypoxia eventually become indistinguishable from each other. The present study tested the hypothesis that reoxygenation in normoxia, following a hypoxic event, results in lower biomarkers of angiogenesis, (i.e., VEGF) and is less injurious to the retina than reoxygenation in Hx between events. To prove our hypothesis, we utilized three clinical-relevant models and compared the results to retinas exposed to normoxia or Hx only.

## 2. Results

### 2.1. Somatic Growth

Table 1 shows percentage change in somatic growth in each oxygen environment. Data are compared to the room air (RA) controls. Animals exposed to Hx (50% O_2_ only), 50–21% O_2_, and 21–12% IH exhibited higher increases in body weight at P14. Conversely, body weight was suppressed in the animals exposed to 50–12% IH. Despite these elevations in body weight, all study animals had lower body length, or shorter stature. Higher body weight with short stature indicates increased body fat or edema. Brain to body weight ratios were lower in the study groups, achieving statistical differences from RA in the 50–12% and 21–12% IH groups. Lung to body weight ratios were lower with Hx exposure. At P21, following 1 week of reoxygenation in RA, all study animals exhibited higher body weight. Body length rebounded in the Hx group and remained suppressed in the 50–12% IH group. Brain to body weight ratios were lower in all groups at P21, and lung to body weight ratios were lower in all groups except the 50–12% group. Significant differences between the 50–12% and 21–12% IH groups persisted at P21.

### 2.2. Eye Opening

Table 2 shows the cecal period (conception to eye opening), which represents retinal neural maturation. Eye opening in rats generally occurs by P14. We examined both eyes to determine whether one or both eyes were affected. Retinal neural maturation was significantly delayed in all study animals, but the group exposed to 50–12% IH was most affected, with less than 20% of animals having a prolonged cecal period. Exposure to 21–12% IH resulted in a greater percentage of rats having a shorter cecal period compared to the animals exposed to 50% O_2_ with or without IH, suggesting that Hx itself is a major deterrent for maturation of the retinal neural circuitry.

### 2.3. Retinal Vascular and Astrocyte Integrity

**Figure 1:**
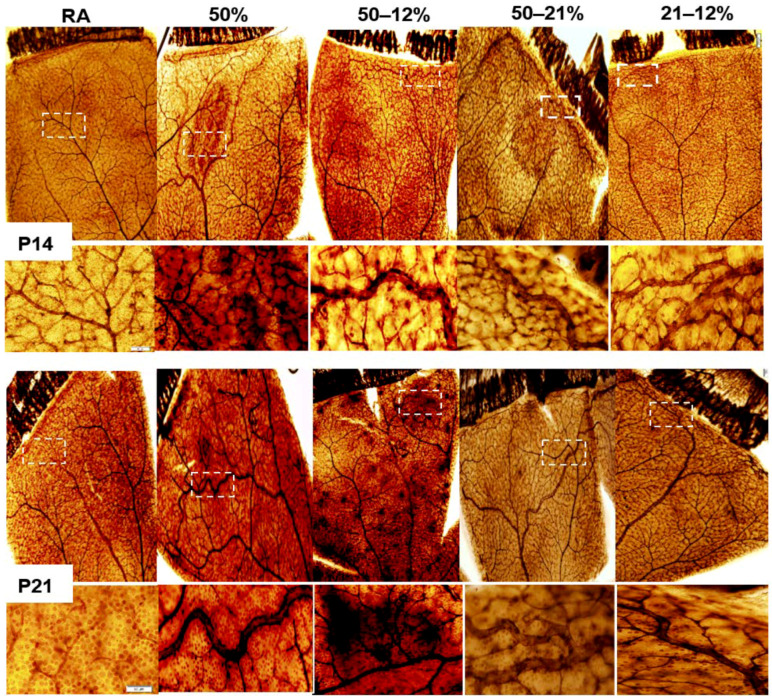
Representative retinal flatmounts demonstrating the effects of neonatal IH paradigms on retinal vascular integrity at P14 and P21. Retinas were stained with ADPase. Images are 10× magnification and the scale bar is 100 µm. White dotted line boxes indicate higher magnification (20×, scale bar 50 µm) images in the panels below.

Figure 1 shows retinal flatmounts stained with ADPase at P14 (upper panel) and P21 (lower panel). At P14, exposure to Hx only resulted in vascular tufts at the periphery and hemorrhage. Exposure to 50–12% IH resulted in hemorrhage, vascular tufts, vascular overgrowth at the periphery, and enlarged vessels.

Similar abnormalities occurred in the retinas exposed to 50–21% and 21–12% IH, with reduced severity in the groups exposed to 21–12% IH. At P21, the severity of OIR increased in all study groups with vascular tortuosity, vascular tufts, enlarged vessels, and vascular overgrowth occurring in all study groups. Hemorrhage was most predominant in the group exposed to 50–12% IH.

**Figure 2:**
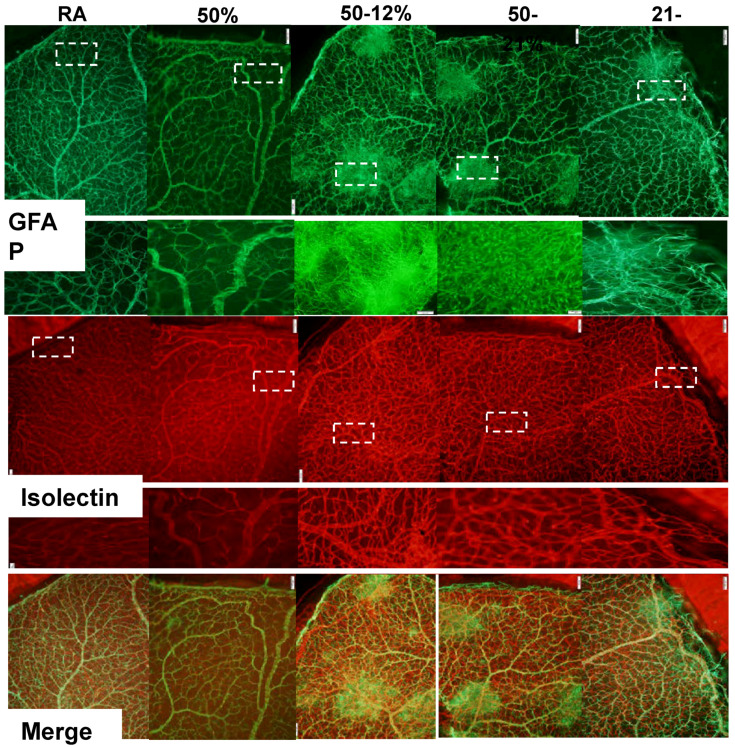
Representative retinal flatmounts demonstrating the effects of neonatal IH paradigms on astrocyte and retinal vascular integrity at P21. Astrocytes and Müller cells were stained for glial fibrillary acidic protein (GFAP) immunoreactivity (green), and retinal vasculature was stained with isolectin B4, a biomarker for endothelial cells (red). Merged images are presented in the bottom panel. Images are 10× magnification and the scale bar is 100 µm. White dotted line boxes indicate higher magnification (20×, scale bar 50 µm) images in the panels below the GFAP and isolectin images.

One of the best-known biomarkers for astrocytes and activated Müller cells is glial fibrillary acidic protein (GFAP), the chief constituent in astrocytes. Astrocytes are found only in the NFL/GCL, they exist proximal to the inner retinal vasculature, and provide guidance cues and structure to the retinal vessels. Isolectin B4 is a specific endothelial cell marker that labels blood vessels. Figure 2 represents retinas stained for GFAP (green) and isolectin B4 (red) at P21. The bottom panel represents the merged images. Data show major disturbances in the astrocyte template and increased vascular abundance, vascular overgrowth, vascular tufts, and enlarged vessels at the periphery in all study groups compared to normoxic age-matched controls.

WimRetina quantification of the retinal vasculature (Table 3), shows significant increases in vascular density, total vascular area, number of branching points, number of segments, and mean segment length in the 50–12% group at P21. Increases in total vascular area and number of branching points occurred with 21–12%, and increases in vascular density, total vascular area, number of branching points, and mean segment length occurred with 50–21%. Of the IH groups, exposure to 21–12% resulted in lower vascular density compared to 50–12%. No significant differences were detected with 50% O_2_ despite higher numbers.

### 2.4. Retinal Morphometry

**Figure 3:**
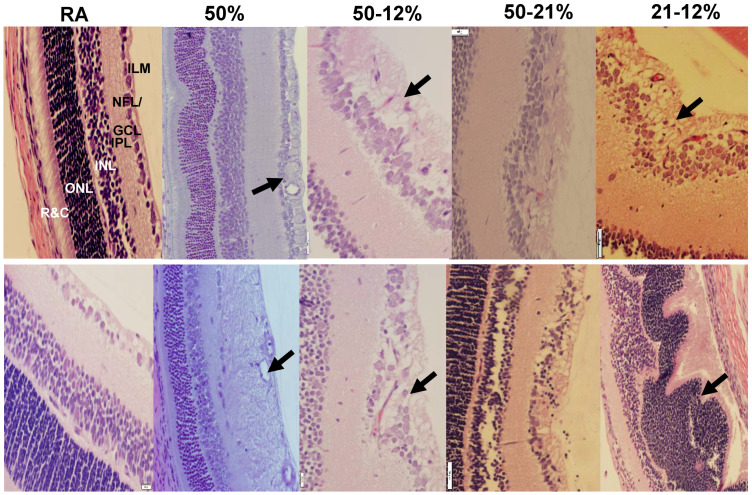
Representative H&E images of retinas showing the effects of neonatal IH on retinal layer integrity and number of endothelial cells in the nerve fiber layer (NFL)/ganglion cell layer (GCL) at P14 (upper panel) and P21 (lower panel). The layers are identified in the RA panel at P14, which is presented as 20× magnification (scale bar is 50 µm) for identification of the retinal layers. All other images are 40× magnification (scale bar is 20 µm). ILM (inner limiting membrane); IPL (inner plexiform layer); INL (inner nuclear layer); ONL (outer nuclear layer); and R&C (rods and cones).

Figure 3 shows the H&E stained retinal layers at P14 (upper panel) and P21 (lower panel). The RA control group at P14 is presented at 20× magnification for identification of all retinal layers. All other images are presented at 40× magnification. The images show widening of the NFL/GCL, increased number of cells, disruption of the ILM, and violation of the vitreous fluid in all study groups at P14 and P21 (arrows). Exposure to 50% O_2_ resulted in abnormalities in the ONL and detachment of the rods and cones (R&C, arrows). At P21, thickening of the NFL/GCL and elevations in cell numbers persisted in all study groups. Exposure to 50% O_2_ resulted in enlarged vessels, and major disruption of the NFL/GCL and inner plexiform layer (IPL) layers. Quantitative analysis of the retinal layers (Table 4) resulted in increased overall retinal thickness in all study groups, except the group exposed to 50% O_2_, which showed overall retinal thinning as well as reduced ONL thickness. Exposure to 50–12% IH induced increased retinal thickness in all areas. Exposure to 21–12% IH increased retinal, NFL/GCL, and ONL thickness, while exposure to 50–21% O_2_ increased retinal and NFL/GCL thickness. Tortuosity index and diameter of vessels increased in the 50% O_2_ and 50–12% IH group, exposure to 21–12% IH resulted in higher tortuosity index and venous diameter, and exposure to 50–21% O_2_ increased tortuosity index only (Table 3).

### 2.5. Angiogenesis Biomarkers in the Ocular and Systemic Compartments

**Figure 4:**
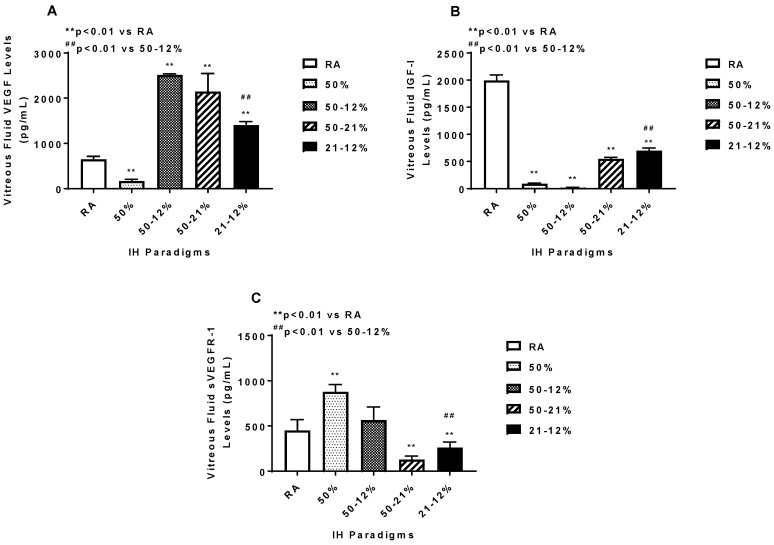
Vitreous fluid vascular endothelial growth factor (VEGF) (**A**), insulin-like growth factor (IGF)-I (**B**), and soluble VEGF receptor (sVEGFR)-1 (**C**) levels in response to variations in oxygen tension. Data are expressed as mean ± SEM (*n* = 6 samples/group). Groups are compared using ANOVA.

The vitreous fluid (VF) is a reservoir for growth factors in the eye. Levels of angiogenesis biomarkers in the vitreous fluid at P21 are presented in Figure 4. No samples were collected at P14, due to low VF volume. VEGF (panel A) in the VF declined with 50% O_2_ and increased in the IH and 50–21% O_2_ groups, although the highest increase occurred with 50–12% and the lowest with 21–12%, resulting in a significant difference between the two groups. In contrast, IGF-I (a permissive factor for VEGF), declined in all study groups compared to RA. The most significant reductions occurred with 50% O_2_ and 50–12% groups, resulting in a significant difference between the two IH groups (panel B). Soluble VEGF receptor (sVEGFR)-1 is a splice variant of the membrane type VEGFR-1. It acts as an endogenous VEGF “trap” and reduces the availability of VEGF to its membrane receptor, decreasing VEGF action. sVEGFR-1 in the VF (panel C) increased with 50% O_2_ and declined in the 50–21% O_2_ and 21–12% IH groups. Of the two IH groups, exposure to 21–12% significantly lowered sVEGFR-1 levels in the VR compared to exposure to 50–12%.

**Figure 5:**
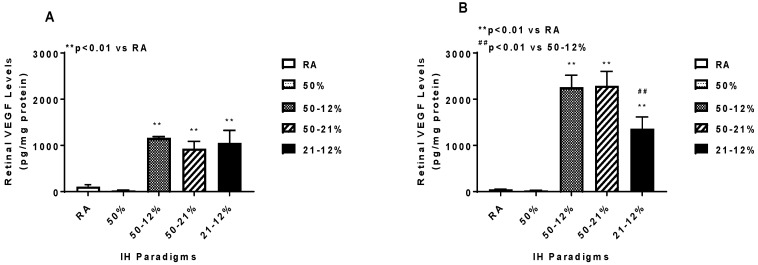

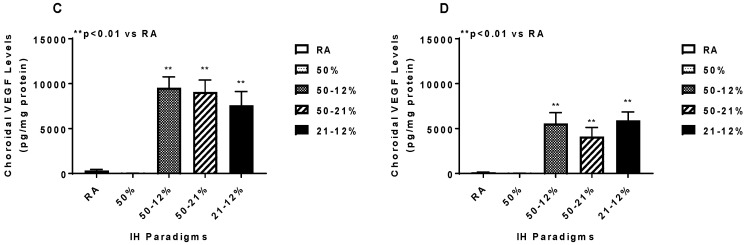
Effects of neonatal IH on retinal (**A**,**B**) and choroidal (**C**,**D**) VEGF levels at P14 (**A**,**C**) and P21 (**B**,**D**). Data are expressed as mean ± SEM (*n* = 6 samples/group). Groups are compared using ANOVA.

Retinal and choroidal VEGF levels are presented in Figure 5. Panels A and C represent data at P14 and panels B and D represent data at P21. Retinal and choroidal VEGF levels increased in the IH and 50–21% groups at P14 and remained elevated at P21, however, the levels at P21 were much higher compared to those at P14. Exposure to 21–12% IH produced significantly lower retinal VEGF levels at P21 compared to 50–12% IH.

**Figure 6:**
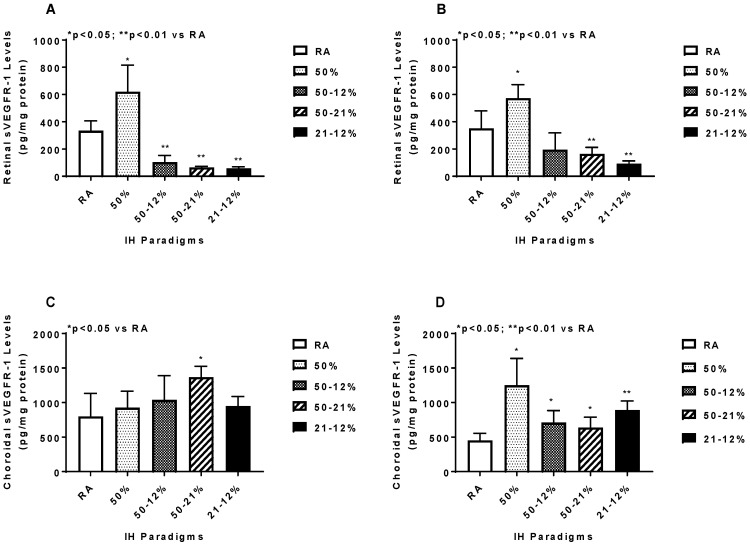
Effects of neonatal IH on retinal (**A**,**B**) and choroidal (**C**,**D**) sVEGFR-1 levels at P14 (**A**,**C**) and P21 (**B**,**D**). Data are expressed as mean ± SEM (*n* = 6 samples/group). Groups are compared using ANOVA.

Retinal and choroidal sVEGFR-1 levels are presented in Figure 6. Retinal sVEGFR-1 increased with 50% O_2_ and declined in all IH and 50–21% O_2_ groups at P14 (panel A) and P21 (panel B). In the choroid, a quite different pattern occurred at P14, with elevations occurring only in the 50–21% O_2_ group (panel C), while at P21, sVEGFR-1 increased in all study groups compared to RA (panel D).

**Figure 7:**
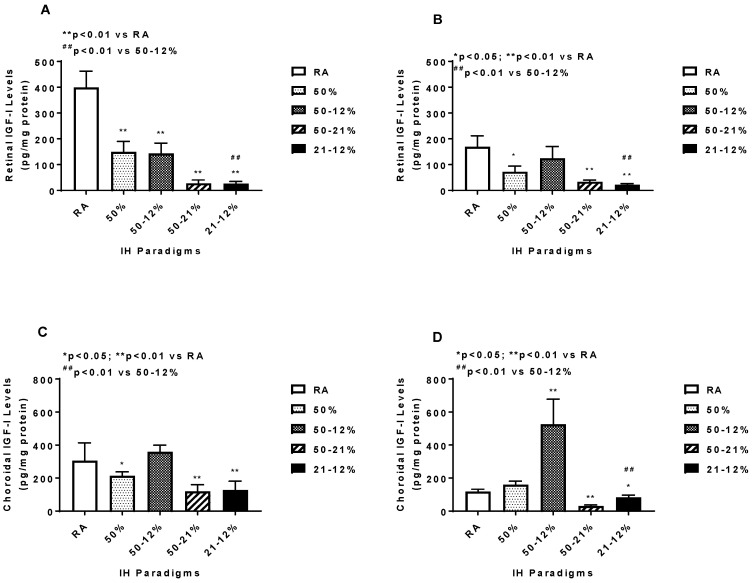
Effects of neonatal IH on retinal (**A**,**B**) and choroidal (**C**,**D**) IGF-I levels at P14 (**A**,**C**) and P21 (**B**,**D**). Data are expressed as mean ± SEM (*n* = 6 samples/group). Groups are compared using ANOVA.

Retinal and choroidal IGF-I levels are presented in Figure 7. Retinal IGF-1 levels declined in the IH and 50–21% O_2_ groups at P14 (panel A) and remained suppressed in the 50% O_2_, 50–21% O_2_, and 21–12% IH groups at P21 (panel B). Exposure to 21–12% IH produced lower retinal IGF-I levels at P14 and P21 compared to exposure to 50–12% IH. In the choroid, IGF-I was similarly suppressed in the 50% O_2_, 50–21% O_2_, and 21–12% IH groups at P14 (panel C). However, at P21, exposure to 50–12% IH resulted in higher IGF-I levels, while the levels remained suppressed with 50–21% O_2_ and 21–12% IH exposure (panel D). This resulted in significant differences between the two IH groups at P21.

Angiogenesis biomarkers in the systemic circulation are presented in Table 5. At P14, VEGF levels declined with 50% O_2_ and increased in all IH and 50–21% O_2_ groups compared to RA. sVEGFR-1 levels were lower in all IH and 50–21% O_2_ groups. Exposure to 50% O_2_ and 50–12% IH resulted in higher IGF-I levels, while exposure to 50–21% O_2_ and 21–12% IH resulted in lower IGF-I levels. At P21, the responses remained sustained, but there was a decline in sVEGFR-1 in the 50% O_2_ group.

## 3. Discussion

The present study employed three clinically relevant paradigms to test the hypothesis that reoxygenation in normoxia, following a hypoxic event, is less injurious to the retina than reoxygenation in Hx between events. The null hypothesis was that there are no differences between the models. These models simulate neonatal IH experienced by ELGANs with low nutritional status, who are at the highest risk for developing severe ROP. For this reason, we monitored growth and systemic growth factors. We noted that body weight was curtailed only in the 50–12% IH group, but elevated in the other study groups with associated reductions in body length. This is suggestive of increased body fat or edema in the higher body weight groups. Elevations in body weight at P21 with deficits in body length at P21 in the 50–12% IH group suggest catchup fat. Correspondingly higher serum IGF-I further implicates its involvement in the catchup fat phenomenon [54,55]. Furthermore, this study showed that all the models resulted in significant retinal abnormalities and elevations in angiogenesis biomarkers, thus allowing us to accept the null hypothesis. This finding was unexpected, considering the shorter range of oxygen variation from 21% O_2_ to 12% O_2_ or from 50% to 21% with recovery in RA, compared to the wide range of 50% O_2_ to 12% O_2_ with no recovery in RA. It is likely that the shorter ranges from 21% O_2_ to either 50% O_2_ or 12% O_2_ did not provide ample recovery time. These data suggest that the immature retina is highly vulnerable to any variations in oxygen, regardless of resolution in RA or Hx between events. Therefore, efforts to curtail these variations should remain a high priority to prevent severe ROP.

Hypoxia is the most potent stimulant of VEGF. Therefore, another surprising finding was that exposure to 50% resolving in RA, with no IH events, caused elevations in ocular VEGF with concurrent retinal damage, suggesting that hypoxic mechanisms were activated in this model. This phenomenon has been referred to as the “normobaric oxygen paradox” which can be explained as the cells interpreting return to normoxia after a hyperoxic event as oxygen shortage or a “relative” hypoxic event, thus inducing hypoxia mechanisms, including hypoxia inducible factor (HIF)_1α_ [56], the transcription factor responsible for upregulation of many genes involved in angiogenesis, cell growth, cell survival, and metastasis [57,58]. Hx induces oxidative damage to various organs, and oxygen sensing by the cell is actually carried out by reactive oxygen species, ROS [59,60,61]. One of the main ROS participating in oxidative damage and lipid peroxidation is hydrogen peroxide (H_2_O_2_). H_2_O_2_ is a principal regulator of the HIF family of transcription factors [62,63] and master regulator of oxidative stress-induced endothelial cell dysfunction. We have shown that H_2_O_2_ is accumulated in the choroid in the 50–12% IH model [50]. It is therefore likely that in the 50–21% model, the “normobaric oxygen paradox” also involves activation of ROS to stabilize HIF and trigger hypoxia in the retina. This mechanism has been reported in critically ill patients [64]. The current findings together with previous reports provide evidence that exposure to hyperoxia with resolution in normoxia is also a potent inducer of hypoxia mechanisms, likely ROS-activated HIF_1α_ [65]. It is likely, that ROS and HIF_1α_ may be the main culprits reacting to any variations in oxygen tension, resulting in elevated ocular VEGF and severe damage to the immature retina, and raises the possibility that therapeutic targeting HIF_1α_ in the setting of neonatal IH may be a viable option for preventing severe OIR. An unexpected finding was the ocular VEGF responses and the vascular density measurements in the Hx only group during the reoxygenation phase at P21. Hx is a known suppressor of VEGF. Therefore, upon recovery in RA at P21, VEGF and retinal vascular density should have been elevated. While we have no definitive explanation for this finding, it is likely that a longer reoxygenation time following chronic Hx may be necessary to mount a relative hypoxia response. It should be noted that high levels of sVEGFR-1—the splice variant of membrane type VEGFR-1 and endogenous inhibitor of VEGF action—was increased in that group. These high levels may be responsible for the low vascular density.

Another interesting finding was the effect of the oxygen variations on retinal astrocyte integrity. Unlike Müller cells, which extend from the INL to outer limiting membranes (OLM) with their end-feet residing in the NFL/GCL, astrocytes are only found in the NFL/GCL [39]. In the normal retina, astrocytes are generally more GFAP positive than Müller cells, however, after injury, both astrocytes and Müller cells rapidly upregulate GFAP. Astrocytes lose their shape and appear irregular and “frayed” [39]. Activated astrocytes are also found to be related to retinal neuronal injury [66], glaucoma [67,68], and increased vascular permeability [69]. We demonstrated a 2.5-fold increase in retinal GFAP using proteomic analysis, validated by Western blot analysis, in our OIR model of 50–12% IH [48], and confirmed by immunofluorescence [45]. In the retina, both astrocytes and Müller cells produce VEGF and appear to play a key role in normal and aberrant retinal vascular formation. Astrocyte and Müller cell reactivity, induced by neonatal IH, may also be responsible for the long-lasting elevations in ocular VEGF derived from these cells, as well as the retinal abnormalities noted in these models. Therefore, curtailing astrocyte and Müller cell reactivity may be important for preventing the VEGF responses and preserving retinal vascular integrity in neonatal IH.

One of the major abnormalities noted in our models is rearrangement of the photoreceptor cells into folds and rosettes, particularly at P21 during the reoxygenation phase [45]. This same phenomenon has been shown in preterm infants and in animal models [44,70,71], and is indicative of apoptosis, degeneration, partial retinal detachment, and loss of retinal pigment epithelium and/or function [72,73]. During normal rat eye development, the photoreceptors and retinal neurons undergo physiologic remodeling and apoptosis, around P10–P25, to reach adult levels [74]. Exposure to neonatal IH may further exacerbate this process and lead to excessive photoreceptor degeneration. Recent studies using optical coherence tomography (OCT) reported increased INL and photoreceptor thickness in preterm infants with ROP who progressed to laser treatment, suggesting that increased retinal thickness [75] is a useful predictor of developing plus disease or stage 3 ROP requiring treatment. In our models, increased retinal thickness occurred in all study groups, and this was predominantly due to widening of the NFL/GCL layer. However, elevated INL and ONL thickness occurred only in the IH groups. It is highly likely that VEGF, a vascular permeability factor, may play a key role in the increased retinal thickness by increasing vascular permeability and edema. Müller cells, the principal glial cells of the retina, also participate in retinal thickness. In the normal retina, Müller cells support neuronal activity and maintain retinal homeostasis. During injury, activated Müller cells, evidenced by increased GFAP expression or gliosis, causes edema via upregulation of aquaporin 4 and downregulation of aquaporin 1 in the ONL, causing breakdown of the blood–ocular barrier and photoreceptor cell death [76].

In conclusion, we have shown, for the first time, that regardless of oxygen tension resolution between events, neonatal IH causes significant deleterious effects on the immature retina. We have also shown that exposure to intermittent hyperoxia with resolution in RA between events results in adverse retinal outcomes similar to intermittent hypoxia with resolution in hyperoxia, confirming the “normobaric oxygen paradox”. These findings address a very important clinical question regarding whether bagging the baby with room air to resolve an IH episode is more beneficial to the immature eye than with oxygen. The data show the outcomes are the same, and this may be due to the high susceptibility of the immature retina to changes or variations in oxygen. Because the retina is an extension of the central nervous system, the deleterious effects in the eye may be indicative of sinister events also occurring in the neonatal brain. Interventions and initiatives to curtail O_2_ variations should remain a high priority. Given the potential role of ROS and HIF_1α_ in the hypoxic responses noted in these experiments, further studies are needed to determine whether timely therapeutic targeting of HIF_1α_ is beneficial to prevent severe OIR in the setting of neonatal IH.

## 4. Material and Methods

### 4.1. Animals

All experiments were approved by the State University of New York, Downstate Medical Center Institutional Animal Care and Use Committee, Brooklyn, NY (Protocol #17-10517, approved on 9 June 2017). Animals were treated humanely, according to the guidelines outlined by the United States Department of Agriculture and the Guide for the Care and Use of Laboratory Animals. Certified infection-free, timed-pregnant Sprague Dawley rats were purchased from Charles River Laboratories (Wilmington, MA, USA) at 18 days gestation. The animals were housed in an animal facility with a 12 h-day/12 h-night cycle and provided standard laboratory diet and water ad libitum until delivery of their pups. All procedures were performed in accordance with the Association for Research in Vision and Ophthalmology statement on the Use of Animals in Ophthalmic and Vision Research and the American Veterinary Medical Association Guidelines for the Euthanasia of Animals.

### 4.2. Experimental Design

Within 2–4 h of birth, newborn rat pups delivering on the same day were pooled (four litters) and randomly assigned to expanded litters of 18 pups/litter. The expanded litter size was used to simulate relative postnatal malnutrition of ELGANs who are at increased risk for severe ROP. The pups were assigned to the following groups: (1) IH (50–12% O_2_) from P0 to P14; (2) IH from P0 to P14 followed by RA from P14 to P21; (3) IH (21–12% O_2_) from P0 to P14; (4) IH from P0 to P14 followed by RA from P14 to P21; (5) 50–21% O_2_ from P0 to P14; (6) 50–21% O_2_ from P0 to P14 followed by RA from P14 to P21; (7) hyperoxia (Hx, 50% O_2_) from P0 to P14; (8) Hx from P0 to P14 followed by RA from P14 to P21; (9) room air (RA) from P0 to P14; or (10) RA from P0 to P21. The RA groups served as controls.

### 4.3. IH Profiles

**Figure 8:**
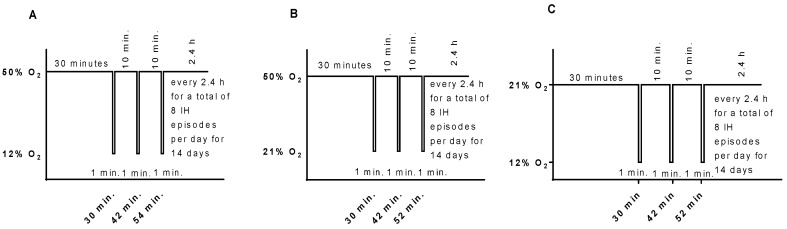
Graphic representation of the neonatal IH paradigms used in this study. Hyperoxia (50% O_2_) following a brief, repetitive hypoxia (12% O_2_) is presented in (**A**). Hyperoxia (50% O_2_) following a brief, repetitive normoxia (21% O_2_) is presented in (**B**). Normoxia (21% O_2_ with brief episodes of hypoxia (12% O_2_) is presented in (**C**).

Animals randomized to oxygen cycling were placed with the dams in specialized oxygen chambers attached to an oxy-cycler (BioSpherix, New York, NY, USA). The animal chambers housed two rat cages, were optimized for gas efficiency, and provided adequate ventilation for the animals in a controlled atmosphere with minimal gas usage. Oxygen content inside the chamber was continuously monitored and recorded on a Dell Computer. Carbon dioxide in the chamber was monitored and removed from the atmosphere by placing soda lime within the chamber. Two IH paradigms were employed: (1) consisting of an initial exposure of Hx (50% O_2_) for 30 min followed by three brief, 1 min, clustered hypoxic events (12% O_2_), with a 10 min recovery in 50% O_2_ between each IH event (Figure 8A). This model of IH was developed and refined in our laboratory. It mimics a preterm newborn with brief, recurrent apnea and desaturations, and simulates babies who are bagged with oxygen during an IH episode. Reoxygenation in 50% O_2_ followed each clustered IH event for 2.4 h for a total of eight clustered IH episodes per day for 14 days, as previously described [45,46,47,48,49,50,51,52,53]; and (2) consisting of an initial exposure of normoxia (21% O_2_) for 30 min followed by three brief, 1 min, clustered hypoxic events (12% O_2_), with a 10 min recovery in 21% O_2_ between each IH event (Figure 8C). This model simulates preterm infants who are bagged with room air during an IH event and recover in normoxia. Reoxygenation in 21% O_2_ followed each clustered IH event for 2.4 h for a total of eight clustered IH episodes per day for 14 days, as previously described [45]. The 50–21% O_2_ profile is presented in Figure 8B. Animals were studied at P14 to determine immediate effects, or at P21, to determine whether the responses resolve following reoxygenation in RA for 1 week. Oxygen saturation was confirmed on a sentinel unanesthetized rat pup from each group using the MouseOx Pulse Oximeter and WinDaq Waveform Browser software (STARR Life Sciences Corp., Oakmont, PA, USA) before and after exposure. The cecal period (conception to eye opening) was recorded to determine the effects of IH on retinal neural maturation [77,78,79]. Percentage changes in body and organ (brain and lungs) weight and body length were assessed at P14 and P21. Blood samples (mixed venous and arterial) were collected for serum levels of angiogenesis factors (VEGF, sVEGFR-1, and IGF-I). Eyes were collected for vascular and astrocyte integrity, retinal layer integrity, retinal morphometry, and angiogenesis biomarkers.

### 4.4. Sample Collection and Processing

For serum biomarkers of angiogenesis, whole blood was collected in Eppendorf tubes with no preservatives and placed on ice for 30 min. The samples were centrifuged at 3500 rpm for 20 min at 4 °C. The resulting serum was transferred to a clean Eppendorf tube and frozen at −20 °C until assay for VEGF, sVEGFR-1, and IGF-I. For retinal and astrocyte integrity, eyes were enucleated and rinsed in ice-cold phosphate-buffered saline (PBS, pH 7.4) on ice. Enucleation was performed with the use of iris forceps and scissors for separation of the eyes from the surrounding connective tissue, nerves, and muscle. Whole eyes were placed in 4% paraformaldehyde on ice for 120 min then flatmounted for ADPase, GFAP, and isolectin B4 staining. For retinal layer integrity and retinal morphometry, whole eyes were fixed in situ in 10% neutral-buffered formalin (NBF). After 2 days, the eyes were enucleated, marked for orientation, placed in 4% NBF, and sent to Histowiz, Inc. (Brooklyn, NY, USA) for tissue processing. Cross-sections (5 µM) were cut through the optic disk and stained with H&E using standard techniques. For ocular biomarkers of angiogenesis, eyes were enucleated as described above and rinsed in ice-cold PBS. The vitreous fluid (VF) was collected by first piercing the eyes and placing them in Eppendorf collection tubes. The eyes were centrifuged at 3000 rpm for 20 min. The vitreous fluid was collected in the outer collection tube. Due to the small VF volume at P14, no samples were collected. The retinas and choroids were then excised under a dissecting microscope (Olympus, USA) and placed in sterile Lysing Matrix D tubes (2.0 mL) containing 1.4 mm ceramic spheres (MP Biomedicals, Santa Ana, CA, USA) and 1.0 mL PBS prior to snap-freezing in liquid nitrogen. Samples were stored at −80 °C until analysis. All samples were analyzed on the same day. On the day of analyses, the tubes were allowed to defrost on ice and placed in a high-speed FastPrep-24 instrument (MP Biomedicals, Santa Ana, CA, USA), which utilizes a unique, optimized motion to efficiently homogenize biological samples within 40 s via multidirectional simultaneous beating of the Lysing Matrix ceramic beads on the tissue. The homogenates were then centrifuged at 4 °C at 10,000 rpm for 20 min. The supernatant was filtered, and the filtrate was used for the assays.

### 4.5. Retinal Flatmounts

Eyes were enucleated and placed in 4% paraformaldehyde on ice for 120 min. The corneas, lens, vitreous, and sclera were removed, and the retinas were cut in quadrants and flattened. Retinal flatmounts for ADPase staining were immersed in 4% PFA overnight at 4 °C. Following several washes in tris maleate buffer (pH 7.2) and incubation in ADPase, retinas were stained with ammonium sulfide, washed, and mounted on slides with phosphate buffered saline (PBS)/glycerin. For Griffonia simplicifolia (GS)-lectin and GFAP staining, retinal flatmounts were fixed in methanol for 20 min, followed by permeabilization and blocking in PermBlock (PBS + 0.3% Triton X-100 + 0.2% bovine serum albumin) in 5% goat serum for 1 h. After washing in PBS/Triton X-100 (TXPBS), flatmounts were then incubated with rabbit GFAP primary antibody (Cell Signaling Technologies, Danvers, MA, USA) overnight at 4 °C. Following several washes with TXPBS, the flatmounts were incubated with Alexa Fluor 488 goat anti-rabbit fluorescent secondary antibodies, and Alexa Fluor 594 Isolectin B4 (ThermoFisher Sci/Life Technologies, Grand Island, NY, USA) overnight at 4 °C. The flatmounts were washed with TXPBS and mounted on slides with *prolong*anti-fade fluorescent mounting media. Images were captured at 20× magnification using an Olympus BX53 microscope, DP72 digital camera, and CellSens imaging software, version 1.18, attached to a Dell Precision T3500 computer (Olympus America, Inc., Center Valley, PA, USA).

### 4.6. Retinal Angiogenesis Quantification

To assess retinal angiogenesis, deidentified ADPase-stained images were uploaded into the Onimagin Technologies website (La Palma, Spain) for Wimasis Software Image Analysis of the vascular area using the WimRetina retinal vessel quantification image analysis software. The four quadrants of each retina were analyzed in a masked manner for vascular density (%, calculated by dividing the number of pixels of the vessels by the total number of pixels of the region of interest), total vascular area, number of branching points (where two or more segments converge), number of segments (number of individual vessel segments), and mean segment length. ADPase-stained retinal flatmounts were used to determine the tortuosity index. A line was traced along the tortuous arteries using the freehand line tool of the Image J software (National Institute of Health, Bethesda, MD, USA) and compared to a straight line traced from the vessel origin at the optic disk to the branch point as previously described [80]. Vessel diameter at the optic disk was quantified using the straight-line tool of the Image J software. The number of endothelial cells present in the nerve fiber layer (NFL)/ganglion cell layer (GCL), the total retinal thickness, and the thickness of the NFL/GCL, inner plexiform layer (IPL), INL (inner nuclear layer), and ONL (outer nuclear layer) were quantified in the H&E stained sections using the count and measure tool of CellSens Dimension software (Olympus America, Inc., Center Valley, PA, USA).

### 4.7. Assay of Angiogenesis Biomarkers

VEGF, sVEGFR-1, and IGF-I levels in the serum, vitreous fluid, and retinal and choroid homogenates were assayed using commercially-available quantikine ELISA kits from R&D Systems (Minneapolis, MN, USA), as previously described [45,47,51,52,53]. All assays were conducted according to the manufacturer’s protocol. All tissue data were standardized using total cellular protein levels.

### 4.8. Total Cellular Protein Levels

On the day of assays, an aliquot (10 µL) of the retinal and choroid homogenates was utilized for total cellular protein levels using the Bradford method (Bio-Rad, Hercules, CA, USA) with bovine serum albumin as a standard.

### 4.9. Statistical Analysis

To determine differences among the group, a test for normality was first conducted using the Bartlett’s test. Normally distributed data were analyzed using one-way analysis of variance (ANOVA) with Dunnett’s post hoc tests to determine differences compared to the RA controls. ANOVA to compare all pairs of groups was also performed with Student–Newman–Keuls, Bonferroni, and Tukey post hoc tests. Non-normally distributed data were analyzed using Kruskall Wallis test with Dunn’s multiple comparison test. Data are presented as mean ± SEM and a *p*-value of <0.05 was considered as statistically significant, using SPSS version 16.0 (SPSS Inc., Chicago, IL, USA). Graphs were prepared using GraphPad Prizm version 7.03 (GraphPad, San Diego, CA, USA).

## Figures and Tables

**Table 1:** Somatic growth.

	RA	50%	50–12%	50–21%	21–12%
***P14:***					
%Change Wt.	216.0 ± 8.6	241.1 ± 13.4 **	198.9 ± 6.6 **	236.1 ± 13.6 **	251.2 ± 12.0 **^#^
%Change Lgth.	72.0 ± 2.2	62.0 ± 2.8 **	45.8 ± 1.4 **	55.3 ± 1.4 **	54.3 ± 2.0 **^##^
Brain/Body Wt. Ratios	0.054 ± 0.0016	0.052 ± 0.002	0.05 ± 0.0014 *	0.051 ± 0.0015	0.048 ± 0.0015 *
Lung/Body Wt. Ratios	0.018 ± 0.0005	0.016 ± 0.0007 *	0.02 ± 0.0009	0.02 ± 0.0007	0.018 ± 0.0007
***P21:***					
%Change Wt.	317.4 ± 17.4	493.9 ± 17.6 **	402.1 ± 10.9 **	443.1 ± 15.5 **	482.2 ± 13.8 **^##^
%Change Lgth.	94.8 ± 4.0	99.1 ± 1.4 **	72.3 ± 2.2 **	94.2 ± 2.0	94.5 ± 3.7 ^##^
Brain/Body Wt. Ratios	0.05 ± 0.002	0.035 ± 0.0009 **	0.042 ± 0.0006 **	0.037 ± 0.0012 **	0.034 ± 0.001 **^##^
Lung/Body Wt. Ratios	0.014 ± 0.0004	0.011 ± 0.0006 **	0.013 ± 0.0004	0.011 ± 0.0006 **	0.01 ± 0.0006 **^##^

(*n* = 18 rats/group; * *p* < 0.05; ** *p* < 0.01 vs. RA; ^#^ *p* < 0.05; ^##^ *p* < 0.01 vs. 50–12% by ANOVA); RA (room air, 21% O_2_); 50% (hyperoxia, Hx); 50–12% (intermittent hypoxia, IH, resolving in hyperoxia, Hx); 50–21%; 21–12% (intermittent hypoxia, IH, resolving in RA).

**Table 2:** Eye opening at P14.

	RA	50%	50–12%	50–21%	21–12%
**Left Eye**	36/36 (100%)	25/36 (69%) **	5/36 (14%) **	25/36 (69%) **	29/36 ^##^ (81%) *^##^
**Right Eye**	36/36 (100%)	26/36 (72%) **	3/36 (8%) **	25/36 (69%) **	27/36 ^##^ (75%) **^##^
**Both Eyes**	36/36 (100%)	20/36 (56%) **	1/36 (3%) **	23/36 (64%) **	27/36 ^##^ (75%) **^##^

All animals were examined at P14 (*n* = 36 rats/group; * *p*< 0.05; ** *p* < 0.01 vs. RA; ^##^ *p*< 0.01 vs. 50–12% by ANOVA). Groups exposed to 50–12% were significantly different compared to all other groups. Groups are as described in Table 1.

**Table 3:** WimRetina quantitation of retinal vasculature at P21.

Vascular Parameters	RA	50%	50–12%	50–21%	21–12%
**Vascular Density (%)**	45.6 ± 0.9	48.4 ± 1.5	53.6 ± 1.9 **	55.2 ± 1.2 **	49.0.2 ± 1.2 ^#^
**Total Vascular Area**	28,525.3 ± 618.4	30,242 ± 1062	33,440.4 ± 1325.2 **	32,104.6 ± 581.5 *	31,514.7 ± 561.0 *
**No. Branching Points**	922.4 ± 38.4	956.8 ± 67.8	1261.8 ± 92.5 **	1145.7 ± 32.7 *	1174.4 ± 48.0 *
**No. Segments**	1549.8 ± 59.7	1631.0 ± 104.4	2091.4 ± 150.9 **	1897.3 ± 75.6	1837.3 ± 109.0
**Mean Segment Length**	16.0 ± 0.4	16.5 ± 0.65	19.2 ± 0.69 **	18.9 ± 0.46 **	17.9 ± 0.49 *

Data are mean ± SD; * *p*< 0.05, ** *p*< 0.01 vs. saline RA; ^#^ *p*< 0.05 vs. 50–12% by ANOVA (*n* = 12 measurements per group). Groups are as described in Table 1.

**Table 4:** Retinal morphometry at P21.

Measurements	RA	50%	50–12%	50–21%	21–12%
Tortuosity Index	1.08 ± 0.01	1.26 ± 0.02 **	1.51 ± 0.01 **	1.36 ± 0.02 **	1.30 ± 0.02 **^##^
Diameter of Arteries	45.2 ± 1.7	37.8 ± 1.3 **	58.6 ± 1.0 **	48.8 ± 1.3	48.9 ± 1.1 ^##^
Diameter of Veins	28.0 ± 0.6	25.0 ± 0.6 **	24.9 ± 0.6 **	27.4 ± 0.6	31.6 ± 0.9 *^##^
Number Cells in NFL/GCL	17.9 ± 0.8	35.9 ± 3.0 **	47.6 ± 2.0 **	56.8 ± 5.1 **	50.9 ± 3.1 **
Total Retinal Thickness (µm)	283.1 ± 14.7	225.7 ± 3.1	468.7 ± 60.2 **	412.7 ± 3.1 **	420.5 ± 7.1 **
NFL/GCL Thickness (µm)	39.9 ± 3.7	56.8 ± 1.6	90.1 ± 15.6 **	69.3 ± 2.5 *	66.3 ± 1.4 *
IPL Thickness (µm)	48.4 ± 6.1	45.8 ± 1.3	103.5 ± 24.5 **	59.7 ± 0.6	69.4 ± 2.7 ^#^
INL Thickness (µm)	70.8 ± 5.7	63.4 ± 0.9	99.5 ± 34.4 *	63.6 ± 1.7	99.0 ± 3.2
ONL Thickness (µm)	90.3 ± 9.2	40.9 ± 1.2 **	151.3 ± 17.5 **	96.4 ± 4.6	134.3 ± 6.3 **

Data are mean ± SD; * *p* < 0.05, ** *p* < 0.01 vs. saline RA; ^#^ *p* < 0.05; ^##^ *p* < 0.01 vs. 50–12%, by ANOVA (*n* = 24 measurements/group); NFL/GCL (nerve fiber layer); IPL (inner plexiform layer); INL (inner nuclear layer); ONL (outer nuclear layer). Groups are as described in Table 1.

**Table 5:** Serum growth factors.

	RA	50%	50–12%	50–21%	21–12%
***P14:***					
VEGF (pg/mL)	40.7 ± 5.4	22.7 ± 1.3 **	256.6 ± 15.3 **	232.4 ± 6.2 **	237.2 ± 6.3 **
IGF-I (pg/mL)	3619.1 ± 166.9	4732.8 ± 158.0 **	6567.0 ± 136.0 **	848.1 ± 160.8 **	779.5 ± 102.6 **^##^
sVEGFR (pg/mL)	615.8 ± 84.1	528.8 ± 82.4	486.5 ± 25.2*	334.9 ± 72.0	452.4 ± 102.0
***P21:***					
VEGF (pg/mL)	45.7 ± 7.7	11.02 ± 0.67 *	211.0 ± 3.8 **	201.5 ± 9.7 **	210.3 ± 13.4 **
IGF-I (pg/mL)	5512.1 ± 212.1	4748.9 ± 123.3 **	7679.3 ± 121.5 **	1248.7 ± 135.5 **	1219.7 ± 121.2 **^##^
sVEGFR (pg/mL)	704.8 ± 62.6	199.6 ± 67.2 **	128.9 ± 22.0 **	334.9 ± 19.7 **	315.9 ± 10.4 **^#^

* *p*< 0.05; ** *p*< 0.01 vs. RA; ^#^ *p*< 0.05; ^##^ *p*< 0.01 vs. 50–12% by ANOVA (*n* = 8 samples/group). Groups are as described in Table 1.

## Abbreviations

ROP	Retinopathy of prematurity
ELGANS	Extremely low gestational age neonates
IH	Intermittent hypoxia
Hx	Hyperoxia
IGF-I	Insulin-like growth factor-1
VEGF	Vascular endothelial growth factor
ECs	Endothelial cells
INL	Inner nuclear layer
NFL	Nerve fiber layer
GCL	Ganglion cell layer
ONL	Outer nuclear layer
ILM	Inner limiting membrane
GFAP	Glial fibrillary acidic protein
P14	Postnatal day 14
P21	Postnatal day 21
sVEGFR-1	Soluble vascular endothelial growth factor receptor-1
O_2_	Oxygen
HIF_1α_	Hypoxia inducible factor-1α
ROS	Reactive oxygen species
H_2_O_2_	Hydrogen peroxide
OIR	Oxygen-induced retinopathy
